# Balancing opportunities and challenges: the double-edged psychological impacts of digital technology empowerment on rural homestay practitioners

**DOI:** 10.3389/fpsyg.2025.1669754

**Published:** 2025-11-24

**Authors:** Zhen Su, Yang Lin, Zhikang Huang

**Affiliations:** 1 School of Business, Guangxi University, Nanning, China; 2 School of Culture and Tourism, Guangxi International Business Vocational College, Nanning, China

**Keywords:** rural homestay practitioner, psychological well-being, self-determination theory, digital technology empowerment, job autonomy

## Abstract

With the increasing prevalence of digital technology, its impacts on worker well-being remain a critical area of inquiry. However, existing research often neglects the dual effects of digital empowerment, particularly in resource-constrained rural contexts. By exploring how digital technology empowerment influences psychological well-being among rural homestay practitioners, this study focuses on the mediating roles of job autonomy and technology anxiety, as well as the moderating role of digital technology self-efficacy. Grounded in self-determination theory (SDT), this study analyzes survey data from 277 rural homestay practitioners in China using structural equation modeling. The results reveal that digital technological empowerment enhances psychological well-being by increasing perceived job autonomy, and likewise negatively affects it by increasing perceived technology anxiety. Additionally, digital technology self-efficacy amplifies the positive effects of empowerment on psychological well-being, but it has a limited impact on reducing technology anxiety. This study extends SDT to the digital technology context and provides a comprehensive understanding of its dual effects on well-being. Practically, the findings offer actionable recommendations for optimizing technology design, strengthening organizational support systems, and fostering user confidence.

## 1 Introduction

The rapid advancement of digital technology is reshaping the global tourism industry, creating new opportunities for businesses to enhance efficiency, expand their reach, and optimize customer experiences (Rodrigues et al., 2023; Moro-Visconti, 2024; Yap et al., 2024). Within this transformative landscape, rural homestays play a pivotal role as a bridge for urban-rural economic integration and are a vital component of the tourism sector. By leveraging digital technology, rural homestays can implement precise marketing strategies, streamline operational management, and improve customer satisfaction (Kapri and Sharma, 2024; Samad et al., 2024). These advancements not only drive the development of rural tourism but also infuse the tourism industry with a distinctive rural culture and set of values. However, rural homestay practitioners often encounter significant challenges in adopting digital technologies due to resource constraints, insufficient digital skills, and management complexities (Samad et al., 2024). Beyond transforming their work practices and operational models, digital technology may also influence their psychological well-being (Johnson et al., 2020).

In China, rural homestay practitioners are currently positioned at the intersection of tradition and innovation. As a country actively promoting rural revitalization, China has implemented a wide range of policies to support digital infrastructure, expand internet access in rural areas, and foster rural entrepreneurship through e-commerce platforms. Initiatives such as Taobao Villages, digital rural pilot zones, and government-backed training programs have significantly reshaped the operational landscape for rural homestay practitioners. However, the introduction of digital tools presents both promising opportunities and inherent challenges (Johnson et al., 2020; Nazareno and Schiff, 2021; Zheng et al., 2025). On one hand, digital technology empowers practitioners by enhancing service quality, streamlining operational processes, and enabling access to a broader customer base, thereby fostering greater work autonomy and a sense of accomplishment (Gerten et al., 2019; Agatić and Kolanović, 2020; Johnson et al., 2020). On the other hand, the complexity and rapid evolution of digital platforms may give rise to technology-related anxiety and psychological stress (Johnson et al., 2020; Marsh et al., 2022; Bhattacharyya, 2023). This dual impact underscores the intricate relationship between digital technology and practitioners' psychological well-being. However, existing research has largely focused on either the positive or negative effects of digital technology in isolation (Marsh et al., 2022; Fleischer and Wanckel, 2024; Liu and Cheng, 2025), leaving the interplay of these dual influences underexplored.

Currently, numerous governments and organizations are actively promoting the empowerment of rural communities through digital technologies to stimulate local economic development (Khan, 2023; Deng et al., 2024; Tu et al., 2025). However, during this transformative process, the well-being of those directly implementing these changes, the rural homestay practitioners, has received little attention. Furthermore, past research on rural homestays has mainly focused on evaluation metrics (Ma et al., 2022; Qiu et al., 2024), consumer motives (Dey et al., 2020), and visitor loyalty (Xing et al., 2022; Dai et al., 2025). Despite the significance of these studies in promoting the development of rural homestays, empirical research on practitioners is still lacking. Particularly under the wave of digital transformation, rural homestay operators face distinct psychological and technological pressures. Recently, scholars have put forward the “digital Empowerment Paradox”, which highlights how digital technologies, while empowering, generate new forms of vulnerability and pressure (Liang et al., 2022; Kokshagina and Schneider, 2023; Zheng et al., 2025). However, the literature has yet to explore how digital empowerment affects their psychological well-being. As the central driving force behind rural tourism, these practitioners are expected to sustain high levels of motivation, creativity, and psychological resilience to operate effectively. Understanding how digital technologies influence their psychological well-being is therefore essential for developing more targeted support policies and practical intervention strategies.

Self-determination theory (SDT) provides a theoretical framework for exploring how digital technology empowerment affects the psychological well-being of rural homestay practitioners through two opposing mechanisms: empowerment and anxiety. SDT emphasizes that when individuals' needs for autonomy, competence, and relatedness are met, they are more likely to experience enhanced intrinsic motivation, leading to improved psychological health and well-being (Gagné and Deci, 2005; Deci et al., 2017). Digital technology empowerment simplifies management processes, improves resource access, and increases information transparency, thus offering rural homestay operators more opportunities for autonomous decision-making and flexible operations (Gerten et al., 2019; Johnson et al., 2020). This increase in perceived autonomy helps satisfy practitioners' basic psychological needs, which in turn stimulates their intrinsic motivation, thereby enhancing their psychological well-being (Deci et al., 2017; Johnson et al., 2020). However, while the introduction of digital technology brings convenience, it can also pose a threat to practitioners' sense of competence, especially when their ability to adapt to technology is limited. Practitioners may experience anxiety due to the complexity of the technology or the pressure to adapt (Bhattacharyya, 2023; Liu et al., 2024). This perceived technological anxiety can undermine their psychological well-being and potentially inhibit the activation of intrinsic motivation (Deci et al., 2017).

Furthermore, individual characteristics can moderate the link between work events and emotional responses (Lazarus, 1991; Chang et al., 2024; Liao et al., 2024). To better understand how individual differences influence these mechanisms, this study introduces digital technology self-efficacy as a moderating variable. In the context of digitalization, individuals' self-efficacy may serve as an important boundary condition in the mechanism through which digital empowerment influences practitioners' psychological well-being. On one hand, high self-efficacy can reduce practitioners' resistance to digital technology, while enhancing their sense of control over it (Galindo-Domínguez and Bezanilla, 2021; Hampel et al., 2024). This increased sense of control significantly boosts their perceived job autonomy, a key factor contributing to higher psychological well-being. On the other hand, high self-efficacy also alleviates the stress and anxiety associated with the complexity of technological tasks (Shu et al., 2011; Makowska-Tłomak et al., 2022), thereby mitigating the negative impact of perceived technology anxiety on practitioners' overall psychological well-being (Saadé and Kira, 2009; Chang et al., 2024). This dual effect highlights the essential role of self-efficacy in fostering a positive relationship between digital technology empowerment and the psychological well-being of practitioners. Examining digital technology self-efficacy as a moderator helps to explain why the effects of digital technology empowerment on psychological well-being may vary among practitioners.

Building on the aforementioned theoretical framework, this study proposes and empirically tests a “dual-impact model” aimed at addressing the following core research questions:

*(i) How does digital technology empowerment influence the psychological well-being of rural homestay practitioners?*

*(ii) What mediating roles do perceived job autonomy and perceived technology anxiety play in this relationship?*

*(iii) How do individual differences in digital technology self-efficacy affect these mechanisms?*

The contribution of this study lies in uncovering the paradox of digital empowerment, namely that digital technologies, while empowering rural homestay practitioners, may also impose psychological burdens. This insight moves beyond prior research that has predominantly emphasized either positive or negative effects, and instead offers a more integrated explanatory framework. By developing a dual-impact model, this study deepens the understanding of the complexity of digital empowerment and extends the application of SDT to the unique context of rural tourism and digital transformation. Moreover, by incorporating digital technology self-efficacy as a moderating factor, the study highlights the critical role of individual differences in shaping responses to the paradox of digital empowerment, thereby opening new perspectives for future research on digitalization and psychological well-being.

## 2 Review and hypothesis

### 2.1 The psychological well-being of rural homestay practitioners in the context of digitalization

Psychological well-being is a multidimensional concept that encompasses an individual's emotional, mental, and social health (Ryff, 1989; Haver et al., 2015). It is crucial for life satisfaction and overall functioning (Ryff, 1989). Psychological well-being is crucial for rural homestay practitioners, as they face not only limited resources but also significant operational pressures and challenges in rural areas (Tu et al., 2025). These practitioners face unique challenges, such as managing customer expectations, maintaining business operations, and navigating economic fluctuations. In recent years, digital technologies have become an indispensable part of rural tourism development, significantly enhancing product visibility, customer engagement, and operational efficiency. However, the increasing reliance on digital tools also brings new challenges that could affect the psychological well-being of these practitioners (Johnson et al., 2020).

In the context of digitalization, the impact of technology on rural homestay practitioners' psychological well-being is complex, functioning as a “double-edged sword”. On one hand, digital technologies can enhance perceived work autonomy by enabling greater flexibility and control over business operations (Gerten et al., 2019; Liu and Cheng, 2025). On the other hand, these same technologies can create perceived technology anxiety (Bhattacharyya, 2023; Yin et al., 2024), as practitioners may feel overwhelmed by the competency demands of digital monitoring, online reputation management, and adapting to new tools. This dual impact reflects the interplay of positive and negative effects, with individual factors playing a significant role in determining outcomes.

**Figure 1:**
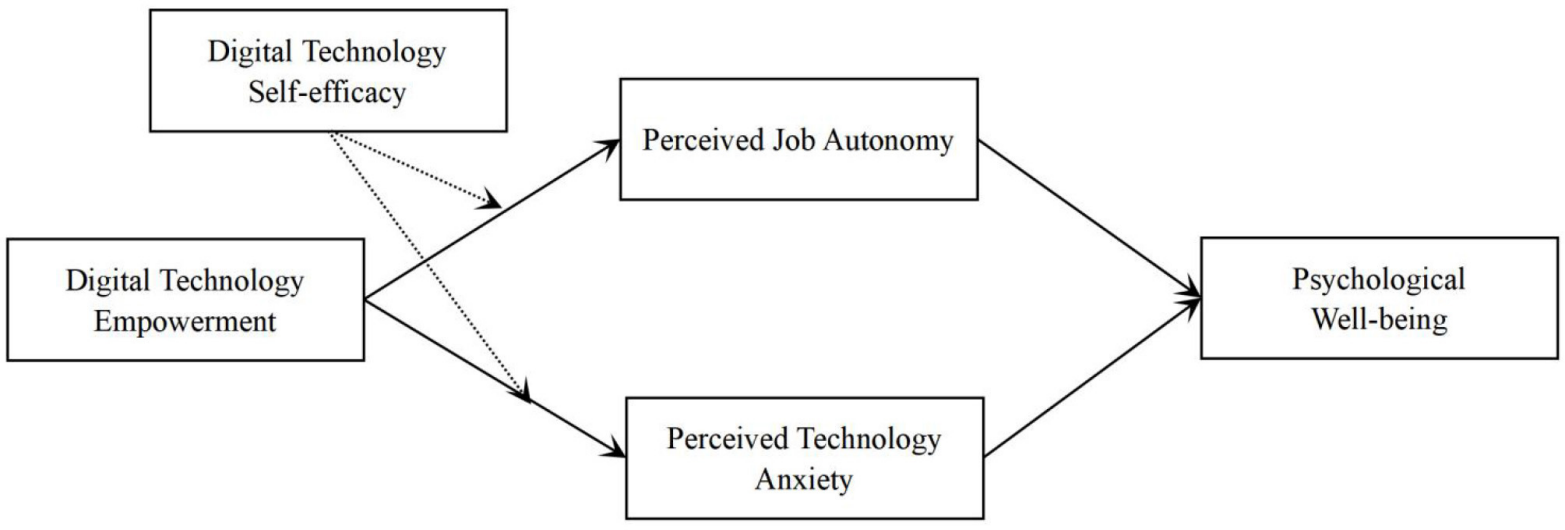
Theoretical model of this study.

Recently, an increasing number of scholars have called for research on the hybrid effects brought about by digital technologies, rather than viewing their positive or negative impacts in isolation (Liang et al., 2022; Troisi et al., 2022; Dong et al., 2024; Zheng et al., 2025). Based on previous studies, this research argues that the introduction of digital technology does not simply produce a one-way effect. Instead, it exhibits a “double-edged sword” nature through different mediating mechanisms (Liang et al., 2022; Dong et al., 2024; Zheng et al., 2025). The purpose is to go beyond the limitation of a purely negative perspective and propose a more dialectical and comprehensive framework. Therefore, when constructing the model, this study does not assume a direct relationship between digital empowerment and psychological well-being. Instead, it introduces two psychological mediators, namely perceived work autonomy and perceived technostress, to explain how digital technology indirectly influences practitioners' well-being through both positive and negative psychological pathways. Figure 1 presents the theoretical model of this study.

### 2.2 The positive mediating role of perceived job autonomy in the relationship between digital technology empowerment and practitioners' psychological well-being

Digital technology empowerment refers to the process of integrating modern digital technologies into operations to enhance individual or organizational efficiency, decision-making capabilities, as well as organizational connectivity and resource integration (Han et al., 2023; Chen Y. et al., 2024). In the rural homestay industry, the empowerment of digital technology is particularly critical due to the common challenges of limited resources and decentralized management. This study suggests that digital technology empowerment can have a positive impact on practitioners' perceived job autonomy. Digital technologies not only reduce the workload of practitioners but also provide them with greater autonomy and flexibility in decision-making (Johnson et al., 2020; RoŽman et al., 2023; Moro-Visconti, 2024). For instance, using digital platforms to conduct real-time data analysis and manage customer feedback allows practitioners to better understand market demands, thereby improving their work efficiency and satisfaction (Rane, 2023). According to SDT, autonomy in the workplace is an important psychological need. Digital technology empowerment can increase practitioners' perceived job autonomy by offering more choices and control. A study by Gerten et al. (2019) and Zheng et al. (2025) also confirmed that digital tools can enhance employees' autonomy in work arrangements. Thus, we propose the following hypothesis:

***H1a:*** *Digital technology empowerment can positively influence practitioners' perceived job autonomy*.

Moreover, perceived job autonomy plays a vital role in the psychological well-being of rural practitioners. Perceived job autonomy refers to an individual's perception of the freedom to make autonomous choices and decisions in their work (Kirmeyer and Shirom, 1986), distinct from objective job autonomy, which refers to the actual decision-making authority and resources provided in the workplace (Hackman and Oldham, 1975; Zheng et al., 2024). The reason for focusing on perceived rather than objective job autonomy in this study is that individuals' perceptions of autonomy have a stronger impact on their psychological experiences and outcomes, such as intrinsic motivation, job satisfaction, and well-being. According to SDT, when individuals perceive higher autonomy, they exhibit stronger intrinsic motivation, which contributes to increased job satisfaction and psychological well-being (Deci et al., 2017). A study on caseworkers found that when employees have greater job autonomy, they are better able to adapt to high-pressure work environments, which reduces emotional exhaustion and improves their mental health (Zhang and He, 2022). The study by Clausen et al. (2022) also confirmed that job autonomy is beneficial to the well-being of workers at all levels. Job autonomy helps workers successfully cope with job demands and other potential stressors in the work environment, thereby promoting individual psychological well-being. Thus, we propose the following hypothesis:

***H1b:*** *Perceived job autonomy has a positive influence on practitioners' psychological well-being*.

Building on the H1a and H1b, we can hypothesize that digital technology empowerment indirectly enhances practitioners' psychological well-being by increasing their perceived job autonomy. Specifically, the tools and platforms provided through digital technology empowerment offer practitioners greater decision-making space and flexibility, which enhances their perceived job autonomy (Gerten et al., 2019). In turn, this perceived autonomy fosters improvements in their psychological well-being (Clausen et al., 2022; Zhang and He, 2022). This reasoning aligns with the core principles of SDT, which asserts that when individuals perceive greater autonomy, their psychological well-being is positively affected. Based on this, we propose the following hypothesis:

***H1c:*** *Perceived job autonomy acts as a mediator in the relationship between digital technology empowerment and practitioners' psychological well-being. Digital technology empowerment can indirectly promote employees' psychological well-being by enhancing their perceived job autonomy*.

### 2.3 The negative mediating role of perceived technology anxiety in the relationship between digital technology empowerment and practitioners' psychological well-being

With the rapid development of digital technologies, these technologies have become crucial tools for enhancing efficiency and innovating services across various industries. However, the widespread adoption of digital technologies may also bring about some negative consequences. In particular, for groups of practitioners with weaker technical backgrounds, the complexity, rapid changes, and constantly evolving demands of digital technologies may create significant psychological pressure, leading to technology anxiety (Johnson et al., 2020; Troisi et al., 2022). Technology anxiety refers to the negative emotions, such as tension, anxiety, and helplessness, that individuals experience when facing new technological tools and operational environments (Meuter et al., 2003; Troisi et al., 2022). These emotions often arise from a lack of understanding of the technology, unfamiliarity with its operation, or concerns about being unable to keep up with technological advancements (Troisi et al., 2022). Yin et al. (2024) suggest that emerging technologies such as artificial intelligence, as external stimuli, can both enhance individual creativity and provoke anxiety when users lack sufficient technical competence. For rural homestay practitioners, digital transformation and intelligent technologies such as smart reservation systems, online marketing platforms, AI-based customer services, and data management tools present similar opportunities and challenges. Because many practitioners have limited access to systematic digital training and hands-on experience, they often experience a sense of competence deficiency when adopting new technologies, and worry their position will be replaced by intelligent technology, leading to technological anxiety (Deng and Liu, 2025). In other words, such anxiety stems from the frustration of basic psychological needs, a mechanism that aligns with the dual influence of technology on creativity and well-being identified by Yin et al. (2024). Previous research has indicated that while digital technology empowerment enhances work efficiency, its complexity and usage thresholds may lead to anxiety, particularly among those less familiar with technology (Nazareno and Schiff, 2021; Bhattacharyya, 2023). Therefore, we propose the following hypothesis:

*H2a: Digital technology empowerment can positively influence practitioners' perceived technology anxiety*.

When individuals experience stress, anxiety, or helplessness, their overall satisfaction with life and work tends to decrease. According to SDT, autonomy, competence, and relatedness are key sources of psychological well-being (Deci et al., 2017; Shir et al., 2019). Technology anxiety, by affecting these fundamental psychological needs, may hurt an individual's mental health. Specifically, technology anxiety may undermine a person's sense of competence, making them feel inadequate in their work (Tams et al., 2018), which in turn affects their work motivation and overall well-being. For example, technology anxiety may lead individuals to feel unable to cope with the application of new technologies, resulting in a decline in self-efficacy, which subsequently affects their emotional well-being and job satisfaction. Some studies on information technology usage found that technology anxiety is positively correlated with emotional fatigue and job burnout (Morska et al., 2022; Marsh et al., 2024), and these negative emotions further impact employees' psychological well-being. Additionally, studies have shown that when individuals feel anxious and frustrated about new technologies, their emotional exhaustion tends to intensify, thereby undermining their psychological well-being (Tawfik et al., 2021; Chen B.-C. et al., 2024; García González et al., 2025). Based on these insights, we propose the following hypothesis:

*H2b: Perceived technology anxiety has a negative influence on practitioners' psychological well-being*.

Building on the H2a and H2b, digital technology empowerment influences practitioners' psychological well-being by increasing their technology anxiety. Digital technology empowerment may lead practitioners to experience anxiety due to unfamiliarity with or inability to adapt to new technologies (Nazareno and Schiff, 2021; Yin et al., 2024; Deng and Liu, 2025), and this technology anxiety can undermine their sense of competence, thereby impacting their psychological well-being (Marsh et al., 2024; García González et al., 2025). According to SDT, an individual's psychological well-being depends on the fulfillment of their needs for competence, autonomy, and relatedness, and technology anxiety can threaten these basic needs, leading to psychological stress and negative emotions, which in turn affect overall mental health. Therefore, we propose the following hypothesis:

*H2c: Perceived technology anxiety acts as a mediator in the relationship between digital technology empowerment and practitioners' psychological well-being. Digital technology empowerment can indirectly undermine employees‘ psychological well-being by increasing their perceived technology anxiety*.

### 2.4 The moderating role of digital technology self-efficacy

Self-efficacy refers to an individual's belief in their ability to perform specific tasks and achieve desired outcomes (Schunk, 1995; Hammer et al., 2021). It plays a central role in motivating behavior, as those with high self-efficacy are more likely to take on challenges, persist in difficult situations, and perform well (Bandura, 1982; Schunk, 1995). In this study, digital technology self-efficacy refers to the belief of rural homestay practitioners in their ability to effectively utilize digital resources and complete related tasks. As digital technologies become increasingly integral to various industries, digital technology self-efficacy becomes crucial in determining how individuals adapt to and utilize these technologies in their work environments.

According to SDT, an individual's psychological well-being is influenced by the fulfillment of three basic needs: autonomy, competence, and relatedness (Church et al., 2013; Shir et al., 2019). Individual characteristics moderating the link between work events and emotional responses (Lazarus, 1991; Liao et al., 2024). When individuals perceive that they have the necessary skills and abilities to perform tasks, they feel a greater sense of competence and autonomy (Bandura, 1982). In the context of digital technology empowerment, individuals with high digital technology self-efficacy are more likely to feel competent in using digital tools (Kim and Lee, 2021), which enhances their sense of autonomy in their roles. One study found that employees who were confident in their ability to use new technologies tended to perceive a greater perceived job autonomy (Moreira-Fontán et al., 2019).

In addition, digital technology self-efficacy also influences how practitioners perceive and respond to stress or anxiety related to digital technologies. According to SDT, individuals with high self-efficacy are more likely to view the adoption of new technologies as an opportunity for personal growth rather than a threat. This mindset allows them to cope with technological changes more effectively, reducing the likelihood of experiencing technology anxiety (Salanova et al., 2002; Lange and Kayser, 2022). When practitioners have a high sense of digital technology self-efficacy, they are better able to cope with the anxiety that new technologies may bring, as they believe in their ability to learn and apply these tools (Deng and Liu, 2025). Research has shown that individuals with lower self-efficacy tend to experience higher levels of stress and anxiety when confronted with new technology, which can impede their adaptation and performance (Chang et al., 2024). Therefore, we propose the following hypotheses:

***H3a:*** *Digital technology self-efficacy positively moderates the relationship between digital technology empowerment and perceived job autonomy. The stronger the practitioners' digital technology self-efficacy, the greater the positive influence of digital technology empowerment on perceived job autonomy*.

***H3b:*** *Digital technology self-efficacy negatively moderates the relationship between digital technology empowerment and perceived technology anxiety. The stronger the practitioners' digital technology self-efficacy, the weaker the positive influence of digital technology empowerment on perceived technology anxiety*.

Moreover, digital technology self-efficacy plays a crucial moderating role in the relationship between digital technology empowerment and practitioners' psychological well-being by influencing the mediating effects of perceived job autonomy and perceived technology anxiety. Psychological well-being is rooted in the fulfillment of autonomy, competence, and relatedness (Church et al., 2013; Shir et al., 2019). While digital technology empowerment offers tools to meet these needs, individual differences in self-efficacy significantly shape outcomes (Chang et al., 2024). Practitioners with higher digital technology self-efficacy feel more capable of using digital tools, which enhances their autonomy at work. This competence allows them to independently manage tasks and make decisions, amplifying the benefits of digital technology empowerment. Thus, individuals with high self-efficacy experience greater job autonomy and satisfaction when using workplace technologies, which in turn improves psychological well-being by fulfilling their autonomy and competence needs. Meanwhile, those with higher self-efficacy feel more confident in managing digital tools, reducing the stress and emotional exhaustion associated with technology use. Studies consistently show that self-efficacy mitigates technology-related stress and anxiety, weakening its negative impact on psychological well-being (Lange and Kayser, 2022; Chang et al., 2024). Therefore, digital technology self-efficacy amplifies the positive mediating role of perceived job autonomy while weakening the negative mediating role of perceived technology anxiety. Based on this, we propose:

***H3c:*** *Digital technology self-efficacy positively moderates the mediating role of perceived job autonomy in the relationship between digital technology empowerment and practitioners' psychological well-being. The stronger an individual's digital technology self-efficacy, the greater the positive mediating effect of perceived job autonomy in this relationship*.

***H3d:*** *Digital technology self-efficacy negatively moderates the mediating role of perceived technology anxiety in the relationship between digital technology empowerment and practitioners' psychological well-being. The stronger an individual's digital technology self-efficacy, the weaker the negative mediating effect of perceived technology anxiety in this relationship*.

## 3 Methodology

### 3.1 Participants and procedures

This study focuses on practitioners working in rural homestays, including both managers and frontline staff. Data were collected through the distribution of paper questionnaires on-site, allowing for direct interaction with participants and obtaining authentic feedback. The survey was conducted in the Guangxi region of China. Guangxi is rich in tourism resources, including unique ethnic cultures, natural landscapes, and traditional villages, making it an ideal area for the development of rural homestays. As part of China's nationwide rural revitalization strategy, Guangxi has seen increasing investment in digital infrastructure and rural tourism initiatives, making it an appropriate empirical context for this study. Moreover, despite these abundant resources, the region remains economically underdeveloped, with many rural areas facing challenges such as limited infrastructure and lower income levels. These structural constraints, combined with active policy support, create a unique environment for observing how digital technologies can empower rural homestay employees and influence their psychological well-being.

To minimize potential common method bias (CMB), some questionnaires incorporated reverse-coded items and the order of items was randomized to assess response consistency. In addition, participant anonymity and confidentiality were strictly maintained throughout the study to encourage honest and open responses. Before data collection, ethical approval was obtained from the Institutional Ethical Review Board, ensuring full compliance with established standards for research involving human participants. The study adhered to the principles of the Declaration of Helsinki, safeguarding participants' rights, privacy, and well-being throughout the research process. All participants provided written informed consent, confirming their voluntary involvement and understanding of the study's purpose and procedures. The formal survey was conducted in January 2025. To ensure a broad and representative sample, 61 rural homestays of various sizes were visited, and a total of 316 questionnaires were collected. After excluding surveys with seven consecutive identical answers or more than five unanswered questions, 277 valid responses were retained. The demographic characteristics of the participants are summarized in Table 1.

**Table 1:** Demographic characteristics of participants.

**Attributes**	**Characteristic**	**Frequency**	**Percentage (%)**
Gender	Male	122	44.04
	Female	155	55.96
Marital status	Single	121	43.68
	Married	143	51.62
	Widowed	5	1.81
	Divorced	8	2.89
Age	18–24 years	77	27.80
	25–34 years	94	33.94
	35–44 years	81	29.24
	≥45 years	25	9.03
Education level	Diploma and below	189	68.23
	Undergraduate degree	76	27.44
	Postgraduate degree	12	4.33
Length of service	<1 year	102	36.82
	1–3 years	72	25.99
	3–5years	55	19.86
	>5 years	48	17.33
Monthly income (RMB)	<2,000	68	24.55
	2,000–3,000	93	33.57
	3,001–4,000	52	18.77
	>4,000	64	23.10
Position	Manager	52	18.77
	Grassroots employee	225	81.23

### 3.2 Measures

This study adopts established scales from internationally recognized academic journals to measure each variable, with adaptive adjustments made according to the research objectives and context. All items were rated using a 7-point Likert scale, where “1” represents “Strongly Disagree” and “7” represents “Strongly Agree”. To minimize translation errors, a back-translation procedure was used to ensure that the main variable scales were accurately adapted to fit the Chinese context, guaranteeing the precision of the questionnaire items. Before the formal survey, a small-scale pilot study was conducted. Based on feedback from the pilot study, revisions were made to address issues such as unclear or difficult-to-understand phrasing, resulting in the final version of the survey questionnaire.

#### 3.2.1 Digital technology empowerment

The digital technology empowerment scale is adapted from Han et al. (2023) and comprises three items designed to evaluate how organizations use digital technologies (e.g., mobile payments, OTA platforms such as Ctrip, social media platforms like TikTok, and online work platforms such as DingTalk) to enhance the operation of rural homestays. A representative item includes: “*Our rural homestay business uses digital technologies (e.g., mobile payments, OTA platforms, social media platforms, online work platforms) to improve customer engagement and online reputation.”* In this study, the scale demonstrated good reliability, with a Cronbach's α of 0.84 and a composite reliability (CR) of 0.90.

#### 3.2.2 Perceived job autonomy

The perceived job autonomy scale is derived from Kirmeyer and Shirom (1986) and consists of seven items to measure rural homestay practitioners' autonomy at work, including decision-making freedom, task execution independence, and job flexibility. A sample item is: “*I had latitude to decide the speed at which I wor*ked.” In this study, the scale's Cronbach's α and CR were 0.93 and 0.94, respectively.

#### 3.2.3 Perceived technology anxiety

The perceived technology anxiety scale is based on Meuter et al. (2003) and contains nine items. To better fit the context of this study, minor modifications were made to the original scale. The items comprehensively assess individuals' anxiety related to digital technology use across these aspects: learning and understanding technology, usage processes, perceived inadequacies, and the consequences of errors. A sample item is: “*I feel apprehensive about using digital technologies.”* In this study, the scale's Cronbach's α and CR were 0.94 and 0.95, respectively.

#### 3.2.4 Psychological well-being

Psychological well-being is measured using the simplified scale developed by Haver et al. (2015), which includes seven items. This scale focuses on evaluating rural homestay practitioners‘ balance between positive and negative psychological states, as well as their overall satisfaction with life. It has been widely applied in human resources research and has demonstrated strong reliability and validity (Summers et al., 2021; Mullens and Laurijssen, 2024). A representative item is: “*I've been feeling optimistic about the future.”* In this study, the scale's Cronbach's α and CR were 0.93 and 0.95, respectively.

#### 3.2.5 Digital technology self-efficacy

The digital technology self-efficacy scale is adapted from (Hammer et al., 2021). Adjustments were made to align the scale more closely with the research focus on digital technologies. This seven-item scale measures practitioners' confidence, comfort, problem-solving skills, and ability to assist others in applying digital technologies. A sample item is: “*I feel good using digital technologies at work to enhance guest experiences and streamline operations.”* In this study, the scale's Cronbach's α and CR were 0.94 and 0.95, respectively.

### 3.3 Data analysis strategy

This study adopts partial least squares structural equation modeling (PLS-SEM) as the analytical approach. The method is chosen because the model involves both mediation and moderation, which adds to its complexity. PLS-SEM is well-suited for exploratory work and the development of new theories. Compared with covariance-based SEM (CB-SEM), it offers greater flexibility for studies at an early stage of theoretical development (Hair et al., 2014). This research focuses on the dual impact of digital technology empowerment on rural homestay practices. PLS-SEM makes it possible to capture these relationships and to test the proposed hypotheses effectively (Hair et al., 2014).

The analysis in this study will be conducted through a series of rigorous steps. First, the reliability and validity of the measurement model will be evaluated, including composite reliability for internal consistency, average variance extracted (AVE) for convergent validity, and the heterotrait-monotrait (HTMT) ratio for discriminant validity. Second, the structural model will be tested to examine the hypothesized relationships, focusing on path coefficients and significance levels, explained variance (*R*^2^), and predictive relevance (*Q*^2^), ensuring the model's appropriateness. Finally, we used importance-performance map analysis (IPMA), a post hoc method, to deepen the understanding of the model's connotations.

## 4 Results

### 4.1 CMB and multicollinearity test

Although procedural controls were implemented to enhance the reliability of the data, it is still impossible to eliminate the potential issues of CMB and multicollinearity. To address these concerns, we employed Harman's single-factor test to examine CMB. The results indicate that the first principal component accounts for 25.46% of the total variance, which is less than 50.00%, suggesting that CMB is not a significant issue (Podsakoff et al., 2003). Moreover, we calculated the variance inflation factors (VIFs) for all independent variables (Hair et al., 2019). All VIF values of the inner model ranged from 1.00 to 1.02, which is below the threshold value of 5.00, indicating that multicollinearity does not pose a problem. These results confirm the reliability of the dataset for subsequent analyses.

### 4.2 Measurement model analysis

To evaluate the measurement model, we assessed reliability, convergent validity, and discriminant validity using widely accepted criteria. First, reliability was examined through Cronbach's α CR values. As shown in Table 2, the results indicated that all constructs had Cronbach's α and CR values exceeding the threshold of 0.70, demonstrating adequate internal consistency (Henseler et al., 2009). Second, convergent validity was assessed by examining the factor loadings, AVE, and CR. All item loadings were significant and above 0.70, while the AVE values for each construct exceeded the recommended minimum of 0.50, confirming satisfactory convergent validity (Hair et al., 2019).

**Table 2:** Construct reliability and validity.

**Constructs**	**Items**	**Loadings**	**Cronbach's α**	**CR**	**AVE**
Digital technology empowerment (DTE)	DTE1	0.88	0.84	0.90	0.75
	DTE2	0.86			
	DTE3	0.87			
Digital technology self-efficacy (DTS)	DTS1	0.86	0.94	0.95	0.72
	DTS2	0.89			
	DTS3	0.75			
	DTS4	0.88			
	DTS5	0.82			
	DTS6	0.86			
	DTS7	0.86			
Perceived job autonomy (PJA)	PJA1	0.86	0.93	0.94	0.71
	PJA2	0.85			
	PJA3	0.89			
	PJA4	0.80			
	PJA5	0.82			
	PJA6	0.83			
	PJA7	0.85			
Perceived technology anxiety (PTA)	PTA1	0.83	0.94	0.95	0.69
	PTA2	0.84			
	PTA3	0.83			
	PTA4	0.85			
	PTA5	0.82			
	PTA6	0.82			
	PTA7	0.83			
	PTA8	0.80			
	PTA9	0.83			
Psychological well-being (PW)	PW1	0.83	0.93	0.95	0.71
	PW2	0.86			
	PW3	0.85			
	PW4	0.86			
	PW5	0.83			
	PW6	0.85			
	PW7	0.83			

The results of discriminating validity are shown in Table 3, the square root of the AVE for each construct was greater than its correlations with other constructs (Fornell and Larcker, 1981). Additionally, the HTMT ratios for all construct pairs were well below the conservative threshold of 0.85, further supporting discriminant validity (Henseler et al., 2009). These results confirm that the measurement model is valid for further analysis.

**Table 3:** Model discriminant validity.

**Constructs**	**Mean**	**SD**	**Fornell-larcker criterion**					**HTMT ratio**				
			**1**	**2**	**3**	**4**	**5**	**1**	**2**	**3**	**4**	**5**
1. DTE	3.93	1.47	**0.87**					/				
2. DTS	3.85	1.68	−0.04	**0.85**				0.05				
3. PJA	3.67	1.53	0.53	0.01	**0.84**			0.59	0.04			
4. PTA	4.24	1.52	0.52	−0.05	0.14	**0.83**		0.58	0.05	0.15		
5. PW	3.72	1.53	0.27	0.03	0.48	−0.10	**0.84**	0.30	0.06	0.51	0.12	/

The bold elements are the square roots of AVE. SD, Standard Deviation; DTE, Digital Technology Empowerment; DTS, Digital Technology Self-efficacy; PJA, Perceived Job Autonomy; PTA, Perceived Technology Anxiety; PW, Psychological Well-being.

### 4.3 Test of the structural model

In this study, PLS-SEM was performed using SmartPLS 4.0. Before testing the structural paths, the explanatory and predictive capabilities of the model were assessed. Specifically, the coefficient of determination (*R*^2^) and cross-validated redundancy (*Q*^2^) values were examined. A higher *R*^2^ value indicates stronger explanatory power, while *Q*^2^ values greater than zero suggest satisfactory predictive relevance (Hair et al., 2019). As presented in Tables 4, 5, the model yielded *R*^2^ values ranging from 0.26 to 0.40 and *Q*^2^ values between 0.06 and 0.37, demonstrating acceptable explanatory strength and predictive validity (Hair et al., 2019). The bootstrapping method was applied with 5,000 random subsamples to estimate both direct and mediating effects.

**Table 4:** Direct and mediated effects test results.

**Hypotheses**	**Path**	**β-values**	***t*-value**	***p*-value**	***VIF***	***R^2^***	***Q^2^***	**Support**
H1a	DTE -> PJA	0.53	11.58	^**^	1.00	0.28	0.27	Yes
H1b	PJA -> PW	0.50	10.44	^**^	1.02			Yes
H1c	DTE -> PJA -> PW	0.27	7.37	^**^				Yes
H2a	DTE -> PTA	0.52	10.30	^**^	1.00	0.27	0.26	Yes
H2b	PTA -> PW	−0.17	3.47	^**^	1.02			Yes
H2c	DTE -> PTA -> PW	−0.09	3.41	^**^				Yes

^**^p < 0.01. ^*^p < 0.05. DTE, Digital Technology Empowerment; DTS, Digital Technology Self-efficacy; PJA, Perceived Job Autonomy; PTA, Perceived Technology Anxiety; PW, Psychological Well-being.

**Table 5:** Moderated effect test results.

**Hypotheses**	**Path**	**β-values**	***t*-value**	***p*-value**	***VIF***	***R^2^***	***Q^2^***	**Support**
H3a	DTS x DTE -> PJA	0.34	5.03	^**^	1.02	0.40	0.37	Yes
H3b	DTS x DTE -> PTA	0.02	0.38	ns	1.02	0.27	0.25	No
H3c	DTS x DTE -> PJA -> PW	0.17	4.47	^**^				Yes
H3d	DTS x DTE -> PTA -> PW	0.00	0.36	ns				No

^**^p < 0.01. ^*^p < 0.05. Ns, not significant; DTE, Digital Technology Empowerment; DTS, Digital Technology Self-efficacy; PJA, Perceived Job Autonomy; PTA, Perceived Technology Anxiety; PW, Psychological Well-being.

As shown in Table 4, all hypotheses of the main model are supported. Digital technology empowerment is positively and significantly correlated with perceived job autonomy (β = 0.53, *t* = 11.58, *p* < 0.01), supporting H1a. Perceived job autonomy positively influences the psychological well-being of rural homestay practitioners (β = 0.50, *t* = 10.44, *p* < 0.01), supporting H1b. Perceived job autonomy plays a significant mediating role between digital technology empowerment and practitioners' psychological well-being (β = 0.27, *t* = 7.37, *p* < 0.01), supporting H1c. Additionally, digital technology empowerment is positively and significantly correlated with perceived technology anxiety (β = 0.52, *t* = 10.30, *p* < 0.01), supporting H2a. Perceived technology anxiety negatively influences the psychological well-being of rural homestay practitioners (β = −0.17, *t* = 3.47, *p* < 0.01), supporting H2b. Furthermore, perceived technology anxiety has a significant mediating effect between digital technology empowerment and practitioners' psychological well-being (β = −0.09, *t* = 3.47, *p* < 0.01), supporting H2c. By comparing the two mediating pathways, we find that digital technology empowerment has more positive than negative influences on practitioners' psychological well-being.

Moderated analysis was conducted using SmartPLS 4.0, and the results are presented in Table 5. The findings indicate that the interaction between digital technology self-efficacy and digital technology empowerment not only has a significant positive direct effect on perceived job autonomy (β = 0.34, *t* = 5.03, *p* < 0.01), but also positively influences practitioners' psychological well-being through perceived job autonomy (β = 0.17, *t* = 3.36, *p* < 0.01), supporting H3a and H3c. However, the interaction between digital technology self-efficacy and digital technology empowerment does not have a significant effect on perceived technology anxiety. Similarly, the interaction does not influence practitioners' psychological well-being through perceived technology anxiety. Thus, H3b and H3d are not supported.

### 4.4 IPMA

To deepen the understanding of the model's implications, we employed IPMA as a post-hoc approach within the PLS-SEM framework. This method is particularly well-suited for our study as it extends the analysis beyond traditional path coefficients by integrating the dimensions of importance (total effects, including both direct and indirect impacts) and performance (average latent variable and indicator scores) (Slack, 1994; Hair et al., 2014). This dual perspective enables an identification of key constructs and actionable insights, even in models where direct effects are limited.

Table 6 presents the IPMA results, revealing that perceived job autonomy exhibits the highest importance (0.50), making it a critical driver of psychological well-being, whereas perceived technology anxiety negatively impacts psychological well-being (-0.17). Digital technology empowerment and digital technology self-efficacy show lower importance, with total effects of 0.15 and 0.03, respectively. On the performance dimension, perceived technology anxiety has the highest score (52.93), followed by digital technology empowerment (48.87), digital technology self-efficacy (47.31), and perceived job autonomy (44.60).

**Table 6:** IPMA for performance impact.

**Constructs**	**The total effect of the construct performance impact (importance)**	**Index values (performance)**
DTE	0.15	48.87
DTS	0.03	47.31
PJA	0.50	44.60
PTA	−0.17	52.93

DTE, Digital Technology Empowerment; DTS, Digital Technology Self-efficacy; PJA, Perceived Job Autonomy; PTA, Perceived Technology Anxiety; PW, Psychological Well-being.

**Figure 2:**
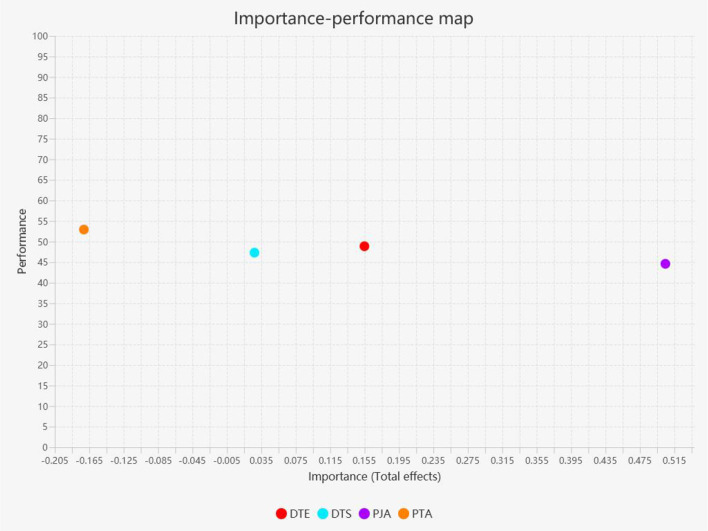
IPMA for PW.

Figure 2 offers a visual representation of the IPMA results for psychological well-being. The x-axis denotes importance (total effects), while the y-axis indicates performance scores. Constructs such as perceived job autonomy, positioned on the far right of the importance axis, underscore their substantial contribution to psychological well-being. In contrast, digital technology self-efficacy is characterized by both low importance and performance, suggesting a lesser priority for immediate interventions. This analysis highlights the need to prioritize improvements in perceived job autonomy while also identifying areas where performance, such as in digital technology empowerment, could be enhanced to strengthen overall outcomes.

## 5 Discussion

This study examines the dual effects of digital technology empowerment on the psychological well-being of rural homestay practitioners, focusing on the mediating roles of perceived job autonomy and perceived technology anxiety, along with the moderating effect of digital technology self-efficacy. The results support most of the hypotheses and provide comprehensive insights into how digital technologies influence well-being in the context of rural tourism.

This study finds that the empowerment provided by digital technology, through tools and platforms that enhance operational control, significantly increases job autonomy among rural homestay practitioners, thereby improving their psychological well-being. Specifically, digital technology provides practitioners with greater control and flexibility, enabling them to make independent decisions and manage daily operations (Liu and Cheng, 2025; Zheng et al., 2025), which boosts job satisfaction and mental health. This finding aligns with SDT, which posits that satisfying the need for autonomy enhances intrinsic motivation and psychological well-being, as observed in the increased job satisfaction and mental health of rural homestay practitioners (Deci et al., 2017). In the context of rural homestay practitioners, digital technology empowerment allows them to access market information and customer feedback, which enables them to adapt their business strategies more effectively, thus increasing their sense of control and decision-making flexibility. This finding is consistent with the work of Gerten et al. (2019) and Fleischer and Wanckel (2024), who found that digital tools significantly increase job autonomy in similar contexts, further enhancing job satisfaction and psychological well-being.

However, this study also reveals the downside of digital technology empowerment, as it significantly increases technology anxiety among practitioners, which in turn negatively impacts their psychological well-being. In the rural homestay industry, practitioners often lack sufficient training and support, leading to anxiety when confronted with digital tools, which undermines their mental health. The findings of this study suggest that although digital technology empowerment enhances work efficiency, the complexity and demands for adaptation can create a psychological burden, which diminishes the positive effects (Yin et al., 2024). This result is consistent with the studies by Meuter et al. (2003) and Tams et al. (2018), who emphasized the correlation between technology anxiety, work stress, and emotional burnout, especially during digital transformation. Marsh et al. (2024) further argued that while digital technology improves work efficiency, its complexity can overwhelm employees, leading to technology anxiety. This study empirically confirms that although digital technology empowerment improves work efficiency, the anxiety it induces can weaken these positive effects, especially in environments lacking adequate training and technical support.

The study also found that digital technology self-efficacy moderates the effect of digital technology empowerment on job autonomy and psychological well-being to some extent. Specifically, when practitioners have higher digital technology self-efficacy, they are more likely to benefit from digital technology empowerment, experiencing increased job autonomy, which subsequently improves their psychological well-being. This finding partially aligns with the work of Lange and Kayser (2022) and (Galindo-Domínguez and Bezanilla 2021), who found that self-efficacy can reduce employees' anxiety and pressure when facing technological challenges. While this study confirms that digital technology self-efficacy enhances the positive effect of empowerment on job autonomy and well-being, it does not significantly reduce technology anxiety. This suggests that such anxiety may stem from structural issues, like system complexity, inadequate training, or poor infrastructure, rather than from a lack of personal confidence (Tawfik et al., 2021). Even individuals with high self-efficacy may feel anxious when external conditions remain unstable or unsupportive. Additionally, persistent uncertainty and generational gaps in digital literacy may also contribute to anxiety, highlighting that self-efficacy alone is not sufficient to buffer all negative emotional responses. Older practitioners, for instance, may experience lingering discomfort despite feeling confident in their ability to learn, due to broader concerns about the pace of technological change or fear of obsolescence (Kim et al., 2023). Reducing technology anxiety may thus require not only individual-level interventions but also systemic improvements in digital design and organizational support.

### 5.1 Theoretical implications

#### 5.1.1 Dual-pathway model and overall effects

To begin with, this study examines how digital technologies, artificial intelligence, and automation impact worker well-being (Johnson et al., 2020; Liu et al., 2024), addressing the theoretical gap in understanding the interplay of positive and negative effects of digital technology on worker well-being. By constructing a “dual-impact model of digital technology empowerment,” this study explores two mechanisms: the empowerment pathway (enhancing job autonomy) and the anxiety pathway (inducing technology anxiety), revealing the complex effects of digital technology on the psychological well-being of rural homestay practitioners. The results demonstrate that digital technology empowerment significantly promotes well-being by enhancing job autonomy (Deci et al., 2017; Gerten et al., 2019; Clausen et al., 2022). However, the complexity and adaptation stress associated with technology partially undermine this positive impact (Meuter et al., 2003; Bhattacharyya, 2023). Unlike studies that focus on either positive or negative effects in isolation, this research systematically compares the combined pathways. It finds that the positive effects outweigh the negative ones, providing a new theoretical framework and empirical evidence for understanding the holistic impacts of technology on well-being.

#### 5.1.2 Moderating role and boundary conditions of self-efficacy

This study introduces digital technology self-efficacy as a moderating variable, which helps clarify the boundary conditions of digital technology empowerment in practice. The results show that self-efficacy significantly enhances the effect of digital empowerment on job autonomy, underscoring the role of personal agency in enabling autonomy-supportive environments (Galindo-Domínguez and Bezanilla, 2021). However, its moderating effect on technology anxiety is not significant. This suggests that while self-efficacy can strengthen perceived control, it may be insufficient to counteract anxiety arising from structural limitations, such as poor system design, lack of training, or unstable infrastructure (Tawfik et al., 2021). This finding highlights the boundary conditions of self-efficacy and resonates with broader discussions on the interaction between individual psychological resources and external support systems (Lazarus, 1991; Marsh et al., 2024). By focusing on rural homestays as a resource-constrained setting, this study not only validates the positive effect of digital empowerment on well-being but also emphasizes the need to improve contextual and technological conditions in order to fully realize its benefits.

#### 5.1.3 Extending SDT and advancing an interdisciplinary framework

The findings extend SDT by clarifying the empowerment and anxiety pathways of digital technology and, by explicitly linking these mechanisms to the digital empowerment paradox, provide a sharper theoretical distinction between the positive and negative effects of digitalization (Kokshagina and Schneider, 2023). By integrating perspectives from psychology, information technology management, and rural development, it broadens the understanding of how technology empowerment shapes psychological well-being, particularly in resource-constrained and high-stress rural tourism settings (Rodrigues et al., 2023; Deng et al., 2024). Moreover, by highlighting the interaction between individual traits (e.g., digital technology self-efficacy) and contextual factors (e.g., technological complexity and organizational support), the study offers a new lens for examining the double-edged effects of technology (Lazarus, 1991; Marsh et al., 2024; Zheng et al., 2025). This interdisciplinary framework advances understanding of the psychological impacts of digitalization and provides a theoretical blueprint for integrating social, technological, and psychological factors in future research.

### 5.2 Practical implications

#### 5.2.1 Organizational strategies

At the organizational level, platform providers, local homestay associations, and destination management organizations should enhance practitioners' perceived autonomy through thoughtful system and process design. This includes granting more flexible control over pricing, booking rules, and availability settings to reduce their passive dependence on platform defaults. In addition, features such as configurable response windows, offline-friendly interfaces, and error-tolerant workflows can help reduce technology-induced anxiety by easing the pressure of real-time responses and complex operations. Digital training should also move beyond standardized instruction toward hands-on, scenario-based micro-tasks and peer mentoring models, enabling practitioners to build self-efficacy through direct experience and application.

#### 5.2.2 Policy interventions

At the policy level, local tourism, human resources, and technology authorities should work together to establish a more inclusive digital infrastructure and public support system for rural tourism. Targeted interventions may include subsidizing broadband access and device upgrades in underdeveloped regions, providing shared content production services, and establishing public digital assistance hubs. Additionally, encouraging data interoperability between local platforms and major OTAs, along with developing standardized APIs, can significantly reduce the cognitive load caused by fragmented systems. Policy initiatives should also integrate psychological support mechanisms alongside digital empowerment programs to ensure that capacity building addresses both technical and emotional dimensions.

#### 5.2.3 Sustainable empowerment

This study highlights that digital empowerment operates through dual psychological pathways: it can enhance autonomy while simultaneously inducing technology-related anxiety. As such, policymakers and industry stakeholders should move beyond the assumption that more digitalization is inherently beneficial. Instead, a more balanced strategy is needed, one that actively mitigates the psychological burden of digital tools while ensuring that empowerment is genuinely meaningful and sustainable. This includes designing systems that allow for greater operational flexibility, reducing forced real-time engagement, and ensuring user interfaces are accessible to those with limited digital experience. Moreover, capacity-building programs should go beyond technical training to incorporate emotional and psychological support, especially for small-scale operators working under resource constraints and social isolation.

### 5.3 Limitations and future directions

While this study offers valuable insights into the dual effects of digital technology empowerment on psychological well-being and extends theoretical understanding in this domain, several limitations must be acknowledged to contextualize its contributions and guide future research.

First, the cross-sectional design of this study constrains our ability to infer causal relationships among the examined variables. While the proposed model demonstrates theoretical plausibility, the static nature of the data limits the robustness of causal conclusions. Future research should consider employing longitudinal or experimental designs to capture temporal dynamics and causal pathways more accurately. Such designs would allow scholars to observe how digital empowerment and its psychological outcomes evolve, and whether feedback loops, such as between empowerment and self-efficacy, emerge through sustained digital engagement.

Second, the study's sample is limited to rural homestay practitioners within a specific cultural and resource-constrained context. While this focus offers a deep understanding of digital empowerment in grassroots tourism entrepreneurship, it may limit the generalizability of findings. To enhance external validity, future research should aim to conduct cross-industry and cross-cultural validation of the proposed dual-impact model. Examining digital empowerment across different sectors (e.g., healthcare, education, manufacturing) and in diverse cultural contexts (e.g., urban vs. rural, collectivist vs. individualist cultures) will help determine the model's universality or reveal potential context-specific mechanisms.

Third, the reliance on self-reported data may introduce common method bias and social desirability effects. Although measures were taken to ensure anonymity and reduce bias, self-perceptions may not fully capture actual behavioral or emotional responses. Future research should consider incorporating multi-source data collection methods, including in-depth interviews, ethnographic observation, or digital behavioral tracking, to triangulate findings and enhance validity. Additionally, applying advanced analytical tools such as natural language processing, sentiment analysis, or machine learning techniques on behavioral or textual data may offer richer and more objective insights into participants' emotional states and adaptive behaviors.

## 6 Conclusion

This study constructs the “dual-impact model of digital technology empowerment” to unveil the intricate mechanisms by which digital technology empowerment influences psychological well-being. It highlights the mediating roles of job autonomy and technology anxiety, alongside the moderating role of individual digital technology self-efficacy. By extending the application of SDT to the context of digital technologies, this research addresses a significant gap in comprehensively examining both the positive and negative effects of digital technology. Theoretically, it offers a fresh perspective on how digital empowerment impacts individual psychology within specific contexts. Practically, it provides actionable insights for managers and policymakers, including strategies to optimize technology design, enhance technical support, and boost user confidence. However, the reliance on a single-industry sample limits the study's generalization. Future research should validate the model's applicability through cross-cultural and cross-industry studies and explore the long-term dynamic effects of digital technologies on psychological well-being.

## Data Availability

The original contributions presented in the study are included in the article/supplementary material, further inquiries can be directed to the corresponding author.
